# Sindbis Virus Infection in Resident Birds, Migratory Birds, and Humans, Finland

**DOI:** 10.3201/eid1401.070510

**Published:** 2008-01

**Authors:** Satu Kurkela, Osmo Rätti, Eili Huhtamo, Nathalie Y. Uzcátegui, J. Pekka Nuorti, Juha Laakkonen, Tytti Manni, Pekka Helle, Antti Vaheri, Olli Vapalahti

**Affiliations:** * Haartman Institute at the University of Helsinki, Helsinki, Finland; † Helsinki University Central Hospital Laboratory, Helsinki, Finland; ‡ University of Lapland, Rovaniemi, Finland; § National Public Health Institute, Helsinki, Finland; ¶ Finnish Forest Research Institute, Vantaa, Finland; # Finnish Game and Fisheries Research Institute, Oulu, Finland; ** Basic Veterinary Sciences at the University of Helsinki, Helsinki, Finland

**Keywords:** alphavirus, arbovirus, birds, Sindbis virus, viral arthritis, zoonosis, research

## Abstract

Sindbis virus (SINV), a mosquito-borne virus that causes rash and arthritis, has been causing outbreaks in humans every seventh year in northern Europe. To gain a better understanding of SINV epidemiology in Finland, we searched for SINV antibodies in 621 resident grouse, whose population declines have coincided with human SINV outbreaks, and in 836 migratory birds. We used hemagglutination-inhibition and neutralization tests for the bird samples and enzyme immunoassays and hemagglutination-inhibition for the human samples. SINV antibodies were first found in 3 birds (red-backed shrike, robin, song thrush) during their spring migration to northern Europe. Of the grouse, 27.4% were seropositive in 2003 (1 year after a human outbreak), but only 1.4% were seropositive in 2004. Among 2,529 persons, the age-standardized seroprevalence (1999–2003) was 5.2%; seroprevalence and incidence (1995–2003) were highest in North Karelia (eastern Finland). Grouse may contribute to the epidemiology of SINV in humans.

## Abstract 2

Resident grouse may be involved in the epidemiology of SINV in humans.

Sindbis virus (SINV) was first recognized and isolated in 1952 from a pool of *Culex pipiens* and *Cx. univittatus* mosquitoes collected from a village in the Nile River delta in Egypt, after which the virus was named (*1*). SINV, a member of the western equine encephalomyelitis complex of the genus *Alphavirus* in the family *Togaviridae*, is an enveloped virus with a genome of single-stranded, positive-polarity, 11.7-kb RNA (*2*). SINV is present throughout the Old World but has never been found in the New World (the Americas). SINV seropositivity in humans has been reported in various areas, and antibodies to SINV have also been found from various bird (*3*–*5*) and mammal (*6*,*7*) species. The virus has been isolated from several mosquito species, frogs (*8*), reed warblers (*9*), bats (*10*), ticks (*11*), and humans (*12*–*14*).

Despite the wide distribution of SINV, symptomatic infections in humans have been reported in only a few geographically restricted areas, such as northern Europe, and occasionally in South Africa (*12*), Australia (*15*–*18*), and China (*13*). In the early 1980s in Finland, serologic evidence associated SINV with rash and arthritis, known as Pogosta disease (*19*). In 2002, SINV was confirmed as the causative agent of Pogosta disease by isolating the virus from acutely ill patients (*14*). The typical clinical picture of acute Pogosta disease consists of arthritis, itching rash, fatigue, mild fever, headache, and muscle pain (*20*). Since 1974 in Finland, the disease has occurred as epidemics of hundreds or even thousands of patients every seventh year (1974, 1981, 1988, 1995, 2002). Similar disease is also found in Sweden (Ockelbo disease) and Russian Karelia (Karelian fever).

Most clinical cases in Finland are reported during August and September; the ornithophilic late summer mosquito species, *Culex* and *Culiseta*, are presumed to be the primary vectors (*21*). Grouse (*Tetraonidae*) (*4*) and passerines (especially thrushes, *Turdidae*) (*5*,*22*) have been suggested as amplifying hosts for SINV in northern Europe. Grouse are of special interest because they have previously had a 6–7 year population cycle with population declines coinciding with SINV outbreaks (*4*,*23*,*24*). Migratory birds may also play a role in distributing SINV over long distances, as they do with West Nile and avian influenza viruses. Supporting information is provided by the fact that SINV is disseminated over vast geographic areas of Australia with isolates from widely separated locations sharing identical nucleotide sequences (*25*). Studies on antigenic relatedness of alphaviruses have also suggested that progenitor alphaviruses are spread over long distances by birds (*26*). Furthermore, phylogenetic studies have indicated that South African and northern European SINV strains are closely related (*14*,*27*). Therefore, the hypothesis is that migratory birds have carried the virus to northern Europe.

A previous human epidemiologic study on SINV from Finland (*4*) reported an annual incidence of 2.7/100,000 during 1989–1996 and a seroprevalence of 0.6% in women of reproductive age during 1992. In addition, SINV antibodies were found in a small number of game birds and mammals in Ilomantsi, eastern Finland, during 1981–1983 (*4*). We studied the human epidemiology of SINV during an additional 7-year period in Finland and the seroprevalence of SINV in resident grouse. Furthermore, we looked for SINV antibodies in migratory birds arriving in northern Europe. Our aim was to elucidate factors contributing to the epidemiologic pattern of SINV infections in humans in Finland.

## Methods

### National Surveillance for SINV and Testing of Human Serum Samples

We used data reported from January 1995 to December 2003 in our analysis of incidence of SINV in the Finnish population. Since 1995, all Finnish clinical microbiology laboratories have reported laboratory-confirmed (by antibody detection) diagnoses of SINV infection to the National Infectious Disease Registry, maintained by the National Public Health Institute in Helsinki. Most laboratory reporting is done electronically and includes date of specimen collection and patient’s date of birth, sex, and place of treatment. Multiple reports for the same person received within a 12-month period are combined as a single case.

For our analysis of the seroprevalence of SINV in the Finnish population, we used all samples from October 6, 1999, to May 8, 2003, that were tested for SINV antibodies at Helsinki University Central Hospital Laboratory. This laboratory performs >70% of the SINV antibody testing in Finland; other testing is performed by the Department of Virology at the University of Turku. We included only the most recent sample from patients with multiple samples; we excluded samples from those who had acute SINV infection (immunoglobulin [Ig] M positive).

### Testing of Resident Grouse

Blood samples from grouse were collected by hunters from September 10 through October 31, 2003, and the same dates in 2004. The blood was absorbed into small slips of filter paper, dried, and stored individually at –20°C. The age and sex of the grouse were determined by hunters.

### Testing of Migratory Birds

**Figure 1:**
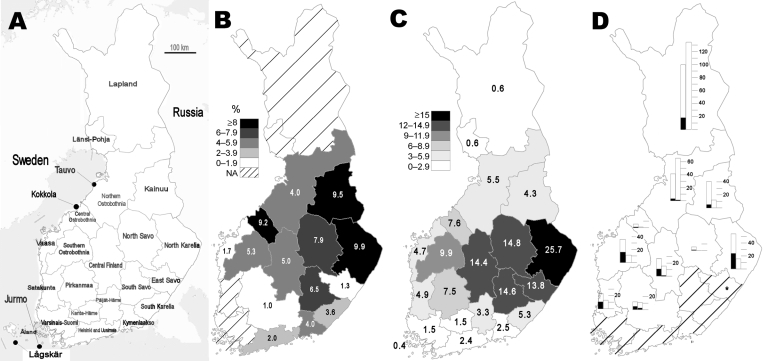
A) Map of Finland with the hospital district divisions. Migratory bird samples were collected from Lågskär, Tauvo, Jurmo, and Kokkola. B) Mean seroprevalence (1999–2003) of Sindbis virus (SINV) infection in human population (according to place of treatment) in the hospital districts of Finland. N/A, not available. C) Average annualized incidence (1995–2003) of SINV infection in human population (according to place of treatment) in the hospital districts of Finland. D) Prevalence of SINV hemagluttination-inhibition antibodies in resident grouse in Finland. The left bars represent the year 2003 and right bars 2004. The bars represent the total number of samples available; the black areas, the number of seropositive samples. *In 2003 in East Savo, the only sample collected was seropositive.

In 2004, blood samples were collected from migratory birds in 2 bird observatories during their spring migration: on Jurmo Island (59°50′N, 21°36′E) on May 18 and 19 and in Tauvo (64°49′N, 24°37′E) May 24–27. In 2005, blood samples were also collected in 2 different bird observatories during the spring migration: on Lågskär Island (59°50′N, 19°55′E) May 22–25 and in Tauvo May 29–31. In addition, migratory bird samples were collected in Kokkola archipelago (63°52′N, 23°4′E) on July 30, 2005 (Figure 1, panel A). Birds were captured with mist nets and identified by certified bird ringers. Blood samples were obtained by absorbing blood from the veins of wings or feet into filter paper slips and then dried. When possible, samples from native birds were also collected into small glass capillary tubes. Samples from migratory birds were collected with the permission of the Animal Experiment Committee of the University of Helsinki (permission no. HY75-04). We used the English and scientific names of birds according to Cramp et al. (*28*).

### Serologic Testing

The human serum samples were examined for SINV IgM and IgG antibodies by enzyme immunoassays (EIA) and for SINV total antibodies by hemagglutination-inhibition (HI) testing. The protocols for EIA (*29*) and HI (*29*,*30*), as well as the diagnostic criteria for acute infection and previous immunity (*29*), have been described. In the incidence analysis, ≈25% of the seropositive diagnoses were made by using EIA at the University of Turku, where HI testing was not used.

The bird samples were screened for antibodies by HI testing. Approximately 1 cm^2^ of each blood-saturated dry filter paper slip was cut into small pieces, and 1 mL of Dulbecco phosphate-buffered saline plus 0.2% bovine serum albumin was added to elute the blood. The resulting dilution corresponded to an ≈1:10 serum dilution (*19*), as verified by parallel titrations with blood (on filter paper) and serum samples from a seropositive person, which were used as controls. A liquid dilution (250 μL) was used for HI analysis.

HI testing was performed with 2-fold dilutions (1:20–1:640). Only titers >40 were considered positive. Performance of the HI technique on bird samples was confirmed by comparison with a set of HI-positive and HI-negative human samples and with neutralization tests (NTs) in which endpoint neutralizing antibody titers inhibiting cytopathic effect on Vero E6 cells were determined ≈65 h after infection. All samples in the subset with an HI titer >40 (n = 7) showed NT titers >20, and all samples in the subset with an HI titer <20 (n = 8) showed NT titers <20. In addition, all samples with borderline HI results were determined by NT to be negative.

## Results

### Human Population

**Figure 2:**
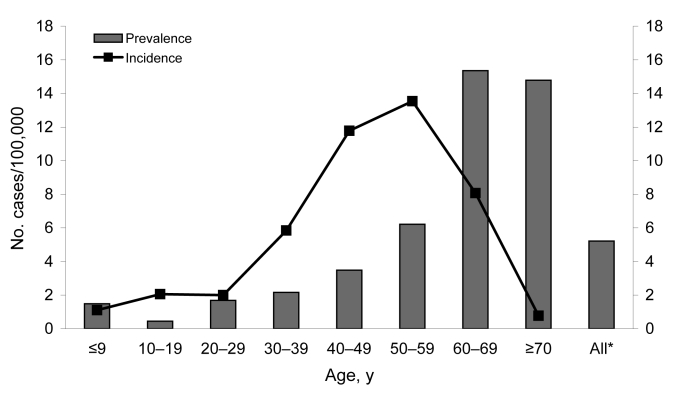
Mean seroprevalence (1999–2003) and average annualized incidence (1995–2003) of Sindbis virus infection in the human population, Finland, according to age groups. *Standardized according to the age distribution of the Finnish population in the respective period.

A total of 2,529 human specimens were included in our analysis. When the data were standardized according to the age distribution of the Finnish population, the estimated seroprevalence was 5.2% (Figure 2). Geographically, the seroprevalence was high in eastern Finland, especially in North Karelia and Kainuu, but also in central Ostrobothnia in western Finland (Figure 1, panel B). Seroprevalence was significantly higher for men (6.0%; mean age of all men studied 41.8 years) than for women (4.1%; mean age of all women studied 44.1 years) (χ^2^ 4,721, p<0.030). Seroprevalence increased with age, reaching 15.4% among persons 60–69 years of age (Figure 2).

**Figure 3:**
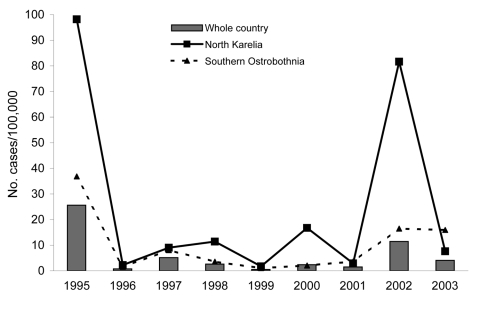
Average annualized incidence of Sindbis virus infection in human population, Finland, 1995–2003.

The incidence of SINV during epidemic years was 25.6/100,000/year in 1995 and 11.5/100,000/year in 2002 (Figure 1, panel B); the average annualized incidence in nonepidemic years (1996–2001 and 2003) was 2.4/100,000. The rates for women and men were 8.7 and 6.6/100,000, respectively. The average annualized incidence was highest (13.5/100,000) among persons 50–59 years of age (Figure 2). Rates were higher for persons in the eastern parts than in the central part of the country and were highest in North Karelia (25.7/100,000; Figure 1, panel C); incidence peaked in North Karelia and in southern Ostrobothnia during the 1995 and 2002 outbreaks (Figure 3). However, a year after the outbreak in 2003, the rates were twice as high in southern Ostrobothnia than in North Karelia (Figure 3).

### Resident Grouse

A total of 340 blood samples were collected from resident grouse in 2003, and 281 samples were collected in 2004 (Table 1; Figure 1, panel D). In 2003, a year after a human outbreak, the total prevalence of SINV HI antibodies in the grouse was 27.4%; in 2004, it declined significantly to 1.4% (Table 1; χ^2^ 76.8, p<0.001). In 2003, the prevalence was high in North Karelia (44%), western Finland (southern Ostrobothnia, Vaasa, and central Ostrobothnia) (44%), and Central Finland (41%), but pronounced also in Lapland in northern Finland (18%) (Figure 1, panel D). Also in 2003, 27.2% of the male grouse and 28.6% of the female grouse were seropositive; in 2004, 2.2% of the males and 1.3% of the females were seropositive (sex was unknown for 132/621 of the grouse). In 2003, 32.0% of the juveniles (born the same year) and 23.3% of the adults were seropositive; in 2004, none of the juveniles and 2.9% of the adults were seropositive (age was unknown for 96/621). The distribution of antibody titers in the positive samples was as follows: 40–120 (32.0%), 160–480 (42.3%), and 640–>1,280 (25.8%).

**Table 1:** Resident birds and Sindbis virus, Finland, 2003 and 2004

Common name	Taxonomic name	2003			2004	
		Samples, no.	Seropositive, %		Samples, no.	Seropositive, %
Capercaillie	*Tetrao urogallus*	67	31.3		41	2.4
Black grouse	*Tetrao tetrix*	144	31.3		84	1.2
Hazel grouse	*Bonasa bonasia*	49	22.4		58	3.4
Willow grouse	*Lagopus lagopus*	80	20.0		98	0
Total		340	27.4		281	1.4

### Migratory Birds

A total of 836 blood samples were collected from migratory birds, of which 806 were collected during spring migration in May 2004 and May 2005. SINV HI antibodies were detected in 3 birds during spring migration: a robin (*Erithacus rubecula*) and a song thrush (*Turdus philomelos*) from Tauvo in 2004, and a red-backed shrike (*Lanius collurio*) from Lågskär in 2005 (Figure 1, panel A; Table 2). HI antibody titers were 240, 120, and 40, respectively. The song thrush was born the previous year; the age of the other 2 positive birds was unknown. SINV RNA could not be detected with reverse transcription–PCR (*20*) from the seropositive filter paper slip solutions. Virus isolation (*14*) from the available whole blood samples (kept at –70°C) from the seropositive robin and song thrush was not successful.

**Table 2:** Migratory birds and Sindbis virus, Finland, 2004 and 2005*

Common name	Taxonomic name	No. samples (no. positive)						
		2004			2005			Total
		Jurmo	Tauvo		Lågskär	Tauvo	Kokkola	
Willow warbler	*Phylloscopus trochilus*	29	105		159	155	3	451
Garden warbler	*Sylvia borin*	1	1		46	2	3	53
Spotted flycatcher	*Muscicapa striata*	7	1		36	3		47
Pied flycatcher	*Ficedula hypoleuca*	7	19		13	7		46
Redstart	*Phoenicurus phoenicurus*	8	16		10	6		40
Whitethroat	*Sylvia communis*	1			34			35
Bluethroat	*Luscinia svecica*	1	30		1			32
Lesser whitethroat	*Sylvia curruca*	4			24			28
Sedge warbler	*Acrocephalus schoenobaenus*		3			5	11	19
Red-backed shrike	*Lanius collurio*	1			8 (1)		5	14
Robin	*Erithacus rubecula*	2	2 (1)		5		2	11
Song thrush	*Turdus philomelos*		11 (1)					11
Blackcap	*Sylvia atricapilla*	4			6			10
Redpoll	*Carduelis flammea*		3		1		2	6
Other (<5 individuals/species)†		5	5		19		4	33
Total		70	196		362	178	30	836

*Sampling was conducted during 2004 in Jurmo and Tauvo and during 2005 in Lågskär Island, Tauvo, and Kokkola. One of the birds sampled was not a migratory bird (willow tit), and 2 birds were not passerines (cuckoo and wryneck). Sindbis virus hemagluttination-inhibition antibodies were detected in 3 (a red-backed shrike, a robin, and a song thrush) of 836 samples. †<5 birds were available from the following species: chaffinch (*Fringilla coelebs*) 4 samples, wood warbler (*Phylloscopus sibilatrix*) 4, icterine warbler (*Hippolais icterina*) 4, reed bunting (*Emberiza schoeniclus*) 3, siskin (*Carduelis spinus*) 3, red-breasted flycatcher (*Ficedula parva*) 2, thrush nightingale (*Luscinia luscinia*) 2, chiffchaff (*Phylloscopus collybita*) 2, willow tit (*Parus montanus*) 1, wryneck (*Jynx torquilla*) 1, cuckoo (*Cuculus canorus*) 1, tree pipit (*Anthus trivialis*) 1, blackbird (*Turdus merula*) 1, grasshopper warbler (*Locustella naevia*) 1, rustic bunting (*Emberiza rustica*) 1, scarlet rosefinch (*Carpodacus erythrinus*) 1, and fieldfare (*Turdus pilaris*) 1.

## Discussion

Our findings show that SINV-seropositive migratory birds arrive in northern Europe during spring migration. Furthermore, after the 2002 outbreak in humans, SINV seroprevalence in grouse decreased markedly between the next 2 consecutive hunting seasons (2003 and 2004). These findings suggest that grouse may be involved in the epidemiology of SINV in humans.

The HI and EIA tests used in this study cross-react only poorly between alphaviruses in different antigenic complexes but may cross-react with other alphaviruses within the same antigenic complex. However, no viruses in the western equine encephalomyelitis complex other than SINV are known to circulate in northern Europe. Seroprevalence of SINV in the human population was analyzed for persons with suspected Pogosta disease; although this sample is not random and was recorded by the place of treatment (not residence), it does provide good representation of different geographic areas.

Although the incidence of SINV infection was higher in women than in men, the seroprevalence was higher in men. This unexpected finding might be explained by the possibility that infected men are more frequently asymptomatic than women, but more investigations are needed. The high seroprevalence but low incidence in Kainuu could be the result of considerable underdiagnosis.

Before 1965, no Finnish persons were found to be SINV seropositive (*31*); however, from 1981 to 1995, seroprevalence of SINV in the Finnish population rose considerably. In 1992, seroprevalence in pregnant women was 0.6% (*4*); in our study, seroprevalence in women 20–39 years of age was 2.3%, which further suggests a continuous increase in the seroprevalence of SINV in Finland. These data suggest that SINV may have been newly introduced to northern Europe during the 1960s to 1970s. As with West Nile virus, a candidate vehicle for the distribution of SINV is infected migratory birds.

Similar to the incidence we found, the incidence of SINV during 1980–1996 was highest in the provinces of North Karelia and Central Finland (*4*). In 2003, however, the incidence was highest in southern Ostrobothnia, possibly reflecting the high immunity to SINV in North Karelia after the 2002 outbreak.

In terms of age, 1 study found SINV seroprevalence in 1,850 hospital patients in Finland to be 19% in those patients <10 years of age (*32*). Our results are in contradiction to this because only 1.4% of persons <10 years of age were seropositive, and seroprevalence increased gradually by age, which we consider a logical finding. In those 60–69 years of age, almost one sixth of the population had immunity to SINV. Considering the high infection rate and that the infection may cause prolonged joint symptoms (*20*,*33*–*35*), even objectively observed by a physician (*36*), the disease is a potential public health concern.

In 1982 in Sweden, most of the 65 serologically diagnosed cases occurred in August. Incidence was highest for men 30–39 years of age and women 50–59 years of age, and prevalence was highest in central Sweden (*37*). Similar temporal and geographic findings were reported for 1981–1987; seroprevalence was 2.5% (*38*).

The last human outbreak in Finland occurred in 2002; ≈600 cases were serologically diagnosed. The next year, the seroprevalence of SINV in grouse was high. One third of the juvenile grouse were seropositive in 2003, which indicates active transmission of the virus to the grouse population that year. A large proportion of these grouse showed markedly high antibody titers. In 2004, the prevalence dropped dramatically; only a few adult grouse were seropositive. These data indicate that grouse become exposed to SINV and that the virus could have an endemic cycle involving grouse. However, other susceptible animals not screened in this study could be involved. Detection of high antibody titers in 2003 implies that the birds have almost certainly been infected and had marked viremia. In addition, the serologic methods that were used in this study (HI and NT) are stringent and can detect only relatively potent antibody responses; the true seroprevalence of the birds may be higher than that reported here.

Until the late 1980s, fluctuation in the grouse population densities in Finland was pronounced and regular, peaking every 6–7 years (*23*,*24*). During the 1970s and the 1980s, the human outbreaks and declines in the grouse population seemed to coincide (*4*). More recently, fluctuation in the grouse population has been less regular with less variation in population density (*39*). Whether the fluctuation of the grouse populations could be a cause or a consequence of the phenomenon behind the 7-year cycle of human outbreaks and whether SINV can be pathogenic to grouse remain unclear. Notably, in 2003, SINV antibodies were detected in various parts of the country, not only in areas where human infection was endemic.

During 1983–1985, Francy et al. (*21*) isolated 14 SINV strains from ≈60,000 female mosquitoes in Sweden. Most were isolated from *Cx. pipiens, Cx. torrentium,* and *Culiseta morsitans,* and seropositive birds were detected during a similar period, which suggests that these species may be important enzootic vectors in a bird–mosquito cycle. In 1983, neutralizing antibodies were not found in the few hundred migratory birds that arrived on the Swedish coast; in the same year, antibody prevalence in nesting birds (residents and migrants) was 3.4% in July and 10% in August (*21*). In the United Kingdom, SINV antibodies have also been demonstrated in resident and migrant birds and in poultry (*3*).

SINV-antibody prevalence in passerine birds sampled in Sweden between June and August during the 1990s (i.e., not during spring migration)—fieldfare (43.3%), redwing (37.0%), and song thrush (22.2%)—was markedly higher than the average (7.7%) of all species studied (*5*). The prevalence was significantly higher for birds sampled after the hatching year (13.9%) than for birds sampled during the hatching year (2.4%).

Of the seropositive migratory birds reported here, robin and song thrush mainly spend the winter in western Europe (some individuals migrate to northwestern Africa) (*28*), and red-backed shrike spend the winter in eastern tropical and southern Africa (*28*). Altogether, 806 of the 836 samples were collected during spring migration in bird observatories, which is usually where the birds first land when they arrive in the country from the sea; the other 30 were collected during midsummer. However, virus infection during the previous year in northern Europe cannot be excluded. Detection of viable SINV in the migrating birds would be the ultimate proof for their involvement in distributing SINV, but that remains to be shown. Extended longitudinal studies are needed to determine whether resident reservoir species are able to sustain SINV cycles endemically in northern Europe or whether the virus must be repeatedly introduced there by migratory birds from the southern hemisphere. Although the similarity of mosquito isolates of SINV from Sweden and Russia in 1980s and from Finnish patients in 2002 (*14*) favors the endemic cycle, larger analyses of SINV strains from Africa and northern Europe (*40*) suggest a continuous importation of South African strains to northern Europe or vice versa.
